# DNA breaks induced by iodine-containing contrast medium in radiodiagnostics: a problem of tungsten?

**DOI:** 10.1186/s41747-018-0050-9

**Published:** 2018-08-15

**Authors:** Mélanie L. Ferlazzo, Clement Devic, Adeline Granzotto, Anne-Marie Charvet, Franck Pilleul, Catherine Colin, Marie-Claude Biston, Aurélie Joubert, Michel Bourguignon, Nicolas Foray

**Affiliations:** 1 Inserm, UMR 1052, Radiobiology Group, Cancer Research Centre, Bâtiment Cheney A, 69008 Rue Laennec, Lyon, France; 2 Inserm, UMR 836, European Synchrotron Radiation Facility, 38042 Grenoble, France; 3 Centre anti-cancer Léon-Bérard, 69008 Lyon, France; 4 Hospices Civils de Lyon, Centre Hospitalo-Universitaire Lyon Sud, 69495 Pierre-Bénite, France; 5 Institut de Radioprotection et de Sûreté Nucléaire, 92260 Fontenay-aux-Roses, France

**Keywords:** Contrast media, DNA, Iodine, Radiation tolerance, Tomography, x-ray computed

## Abstract

Iodine-containing contrast media (ICM) are extensively used to improve image quality and information content in x-ray-based examinations, particularly in computed tomography (CT). In parallel, there is increasing evidence that the use of ICM during CT sessions is associated with deoxyribonucleic acid (DNA) breaks that may influence the estimation of the risks linked to x-ray exposure. Why has iodine been preferred to any other heavy elements to enhance contrast in radiodiagnostics? How to understand such DNA breaks effect? We searched for the answers in the early times of x-ray medical use. It appeared that the maximal ratio between the relative iodine and water mass energy absorption coefficients is reached in the range of 40–60 keV, which defines the energy range in which the dose is preferentially absorbed by ICM. This range does not correspond to the K-edge of iodine but to that of tungsten, the major component of the x-ray tube anode of CT scanners. At such energy, radiolysis of the ICM produces sodium or potassium iodide that prevents a normal DNA breaks repair and influences the individual response to x-ray low-dose. Both contrast enhancement and DNA breaks effect may therefore be caused by tungsten of the anodes of x-ray tubes.

**Electronic supplementary material:**

The online version of this article (10.1186/s41747-018-0050-9) contains supplementary material, which is available to authorized users.

## Key points

- The historical development of ICM has followed an empirical approach.
- The contrast is defined by the ratio iodine/water mass energy absorption coefficients.
- This ratio does not reach its maximal value at the iodine K-edge.
- This ratio reaches its maximal value at the tungsten K-edge.
- Iodine-related contrast is mainly caused by tungsten of x-ray tubes anode.

## Introduction

Iodine-containing contrast media^1^ (ICM) are extensively used to improve image quality and information content in x-ray-based examinations, particularly in computed tomography (CT) [1, 2]. In parallel, there is increasing evidence that the use of ICM during irradiation is associated with the production of additional deoxyribonucleic acid (DNA) breaks (Additional file 1: Table S1). Hence, why has iodine been preferred to any other heavy elements to enhance contrast in radiodiagnostics? How to understand such excess of DNA breaks?

### Iodine as a contrast agent for CT exams: an empirical approach

As a first step, we searched for the answers to these questions in the early times of x-ray medical use.

The principles of contrast enhancement were born with radiology. Even before the first explanation of the photoelectric effect by Einstein in 1905, a number of radiologists developed strange recipes to render radiopaque the organs to be studied. In Vienna, Austria in January 1896 (i.e. some weeks after the Roentgen’s discovery), Eduard Haschek and Otto Theodor Lindenthal [3] injected a mixture made of lime, cinnabar (mercury) and petroleum oil in the amputated hand from the cadaver of an old woman and observed the vessels by radiography. This was considered to be the first angiogram. In Lyon, France, in November 1896, Étienne Destot and Léon Bérard applied solutions containing gold or bronze powder to visualise brain and thyroid vasculature [4]. Other metals such as barium, lead and bismuth were also used for the same purpose [5]. Hence, radiologists intuitively favoured the use of heavy elements as contrast media while iodine was never cited before the 1920s.

Discovered in 1811, iodine was proposed for the treatment of syphilis as a mixture of iodine and potassium iodide in 1831 by Jean Guillaume Auguste Lugol. However, it was William Wallace in Dublin who contributed the most to the development of iodine therapy against syphilis [6]. While the direct curative effect of iodine on syphilis is still debated, the extensive application of iodine therapy in the 1920s, combined with routine x-ray observations, led to the empirical conclusion that urine in the bladder becomes radiopaque in sodium iodide-treated patients; iodine was therefore considered a promising contrast medium [7]. However, sodium iodide was too toxic and was eliminated too slowly [7]. Composed of benzene rings incorporating iodine atoms, the first ICM were lipiodol [7] and uroselectan, which led to the first intravenous urograms [8]. Anaphylactic reactions were reported early and a fierce race to develop safer ICM began in the middle of the 1920s [9].

**Fig. 1:**
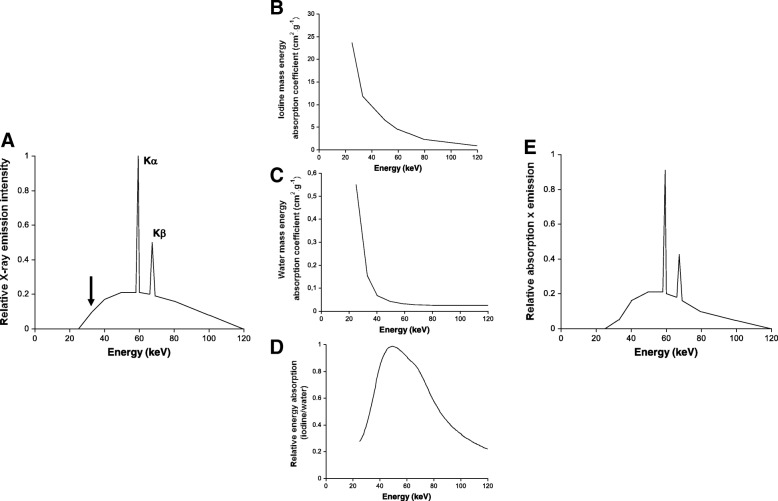
Schematic representation of the x-ray emission and absorption spectra related to the use of ICM during a CT session. **a** Relative emission x-ray spectrum of a typical 120 kVp CT scan. The arrow indicates the 33.2 keV component of the spectrum that would correspond to the K-edge of iodine. Iodine **b** and water **c** mass energy coefficients μ_en_/ρ expressed in cm^2^.g^− 1^ as a function of energy. **d** Relative energy absorption of iodine in water (normalised ratio between the relative iodine and water mass energy absorption coefficients μ_en_/ρ) as a function of energy. **e** Product of the relative emission intensity of a typical 120 kVp CT x-ray tube and the relative absorption of x-ray by ICM

Hence, the use of iodine to enhance contrast during x-ray exams was not led by a documented and logical approach but rather by empirical observations. Interestingly, to date, the atomic properties of iodine are evoked a posteriori to better explain the choice of ICM in contrast-enhanced CT practice. Notably, it is argued that since the most frequent x-ray tubes used in CT deliver a polychromatic 100–140-kVp x-ray spectrum that includes energies of 30–40 keV, it can trigger photoactivation of iodine whose K edge is at 33.20 keV [1]. Such a statement does not stand up to a quantitative and rigorous analysis of physical data. Indeed, the typical x-ray spectrum for CT is composed of a pseudo-bell-shaped curve from 20 to 80, 100, or 120 keV with a maximum around 50 keV. However, at this energy, the relative x-ray emission intensity never exceeds 20%. The iodine K-edge of 33 keV x-rays representing about 10% of emitted x-rays cannot quantitatively explain the contrast enhancement observed with ICM (Fig. 1, Table 1).

**Table 1:** X-ray energy emission and absorption features for tungsten, iodine and water

Energy (keV)	Relative emission intensity of a typical 120-kV CT x-ray tube	Iodine mass energy absorption coefficient μ_en_/ρ	Water mass energy absorption coefficient μ_en_/ρ	μ_en_/ρ (iodine) / μ_en_/ρ (water)	Relative absorption (iodine in water)	Relative emission × absorption
20	0	23.63	0.55	42.96	0.28	0
33 (I K edge)	0.10	11.90	0.1557	76.429	0.49	0.049
40	0.18	9.62	0.06947	138.47	0.89	0.1602
50	0.21	6.57	0.04223	155.576	1	0.21
59.3 (W K_α_ edge)	1	4.58	0.0328	139.634	0.91	0.91
60	0.21	4.52	0.0319	141.692	0.90	0.1911
67.23 (W K_β_ edge)	0.50	3.77	0.0285	132.28	0.85	0.425
80	0.17	2.33	0.02597	89.71	0.57	0.0969
120	0	0.91	0.0265	34.33	0.22	0

μ_en_/ρ are expressed in cm^2^.g^−1^

### When iodine meets tungsten

In addition to the broad polychromatic x-ray spectrum evoked above, two major x-ray peaks are observed, both related to the element that composed x-ray tubes anodes: tungsten (Fig. 1). First chosen by Franz Hanaman and Alexander Just in 1904 for the incandescent lamp because of its high melting point and its ductility, tungsten was introduced in x-ray tube anode by Coolidge in 1906.^2^ The tungsten K_α_ and K_β_ energies are at 59.30 and 67.23 keV, respectively. While relative x-ray emission intensity at 67.23 keV is < 50%, it is maximal (100%) at 59.30 keV, i.e. ten times more than at 33 keV (Fig. 1, Table 1). This x-ray range around 60 keV made x-ray tubes with a tungsten anode particularly suitable for CT. For mammography, for which x-rays do not need to be more penetrating, the anode is generally made of molybdenum or rhodium and its two K-edge energies are at 17.5 and 19.6 keV, and 20.3 and 22.7 keV, respectively.

The conclusion that the most intensively emitted monochromatic energy of polychromatic x-ray CT tubes is 59.3 keV would have remained a simple detail if this energy did not also correspond to the maximal energy absorption of iodine atoms in water. In fact, with regard to contrast enhancement with ICM, two major points have to be considered: (1) the K-edge of iodine atoms at 33.20 keV corresponding to the maximal photoelectric effect; and (2) since ICM are injected in biological tissues, the energy absorption in water must be considered.

The maximal ratio between the relative iodine and water mass energy absorption coefficients μ_en_/ρ is reached in the range of 40–60 keV. Consequently, at 59.30 keV, the contrast obtained with ICM is maximal. Such a property is directly due to the composition of the x-ray tube anode in tungsten. Another element would drastically change the usefulness of ICM (Fig. 1, Table 1).

### The bad and good sides of a coincidence

The contrast phenomenon corresponds to a maximal energy local absorption, i.e. to a higher local dose in comparison to the dose absorbed in the absence of ICM. Hence, while this phenomenon is useful for the aim of diagnosis, such excess energy absorption may be responsible for specific molecular and cellular effects.

In 2005, it was shown that ICM could be photo-degraded (due to radiolysis) at CT scan energies and generate free I^−^ ions that rapidly associate with organic potassium or sodium to give sodium or potassium iodide [10]. In fact, unlike ICM molecules, iodides easily enter into the cell cytoplasm and nucleus, where they can interact with DNA. The dose-dependent production of iodides from ICM inhibits DNA double strand break (DSB) repair by decreasing the DNA-dependent protein kinase activity, an important signalling step required for a normal response to radiation. In parallel, such inhibition may favour error-prone DSB repair pathways, which can increase genomic instability and cancer risk [10]. The observations that the presence of ICM during CT exams leads to DNA or chromosome breaks are not new and this radio-sensitisation effect was first observed in the 1970s with cytogenetics assays [11] (Additional file 1: Table S1). Some authors also reported an excess of DSB in the lymphocytes of patients undergoing CT exams but a potential ICM-specific effect was not evoked [12–14]. The practical consequence of this excess of DSB (that corresponds to an excess of biological dose) suggests that the knowledge of the physical dose, whether assessed or calculated by standard methods, is not sufficient to better estimate the related risks when ICM are present during CT scan exams.

### Influence of individual radiosensitivity

To date, there is an increasing evidence that unrepaired DSB are responsible for cell lethality and tissue radiosensitivity and that misrepaired DSB are linked to genomic instability and cancer proneness [15]. In the low-dose exposure of CT exams, tissue over-reactions are not expected and anaphylactic reactions observed in some patients shortly after ICM injections are not necessarily linked to unrepaired DSB [16]. Conversely, misrepaired DSB contributing to an increased cancer risk represent one major outcome of the ICM effect, even if there is still no consensus about specific misrepaired DSB markers. In addition, at the doses applied during CT exams, some specific effects, such as the low-dose hypersensitivity phenomenon, may lead to both excess of cell death and mutations [17, 18]. Thus, the contrast enhancement obtained by ICM may also strongly depend on individual radiosensitivity, which could explain the discrepancy between different authors about its quantitative contribution to the total dose (Additional file 1: Table S1).

## Conclusion

While the use of ICM in radiodiagnostics was not based on a logical step-by-step approach, their efficiency to enhance contrast may lead to an excess of biological dose. The clinical consequences of such excess are likely to be dependent on the concentration of ICM and on the individual radiosensitivity. Further investigations that would take into account all these factors may be useful for a better estimation of the potential risk linked to contrast-enhanced CT exams.

## Additional file

Additional file 1: **Table S1**. Non-exhaustive list of reports investigating cell survival, chromosome and DNA damage in human cells exposed to radiation in presence of ICM. (DOCX 35 kb)

## Abbreviations

CT: Computed tomography
DNA: Deoxyribonucleic acid
DSB: Double strand break
ICM: Iodine-containing contrast media

## Footnotes

1 According to a recent semantic study [2], we preferred the term ‘iodine-containing’ to ‘iodinated contrast agent’, which has the advantage to keep the commonly accepted ‘ICM’ abbreviation.

2 Until this date, the x-ray tube anode was made by cold metal and not by tungsten that reaches about 3000 °C when x-rays are emitted.
