# Supplementary material for: DNA breaks induced by iodine-containing contrast medium in radiodiagnostics: a problem of tungsten?
Source: Eur Radiol Exp. 2018 Aug 15;2:21. doi: 10.1186/s41747-018-0050-9 (PMC6092269; doi:10.1186/s41747-018-0050-9)
Supplement: Supplementary file 1 — Table S1. Non-exhaustive list of reports investigating cell survival, chromosome and DNA damage in human cells exposed to radiation in presence of ICM. (DOCX 35 kb) [file 41747_2018_50_MOESM1_ESM.docx]

**DNA breaks induced by iodine-containing contrast medium**

**in radiodiagnostics: a problem of tungsten?**

**Additional file 1**

**Table S1: Non-exhaustive list of reports investigating cell survival, chromosome and DNA damage in human cells exposed to radiation in presence of ICM**

| **References** | **X-rays**  **exposure** | **Human cellular model** | **ICM** | **Endpoints and conclusions**  **about the effect of ICM** |
| --- | --- | --- | --- | --- |
| *Cytogenetics* | | | | |
| [1] | Angiography | Lymphocytes | Renografin-76  (diatrizoate) | Excess of micronuclei and  chromosome aberrations observed  *in vivo* |
| [2] | Urography | Lymphocytes | Hypaque sodium  (diatrizoate) | Excess of micronuclei observed  *in vivo* |
| [3] | Angiography | Lymphocytes | Several ICM | Excess of micronuclei observed  *in vivo* and *in vitro* |
| [4] | Urography | Lymphocytes | Diatrizoate and  ioxaglate | Excess of micronuclei observed  *in vivo* |
| *Cell survival* | | | | |
| [5] | 33-70 keV  synchrotron  X-ray | SQ20B tumor cells | Iomeprol | Cellular radiosensitization |
| *DSB repair and signaling pathways* | | | | |
| [6] | CT | Lymphocytes | Iomeron 300  Ultravist 300 | γH2AX foci levels were 30% higher  in CT patients with ICM |
| [7] | Coronary CT  angiography | Lymphocytes | Iomeprol 350 | Lower γH2AX foci levels after sequential  than after helical CT  ICM effect is evoked |
| [8] | CT | Lymphocytes | Untravist 300 | γH2AX foci levels were 58% higher  in CT patients with ICM |
| [9] | CT | Lymphocytes | Untravist 300 | Higher γH2AX foci levels in CT patients  than expected the dose-length product.  Effect of ICM evoked |
| [10] | CT | Lymphocytes | 7 different ICM | γH2AX foci levels were 38% higher  in CT patients with ICM The increase  of DSB is dependent on iodine concentration |
| [11] | CT | Lymphocytes | Ultravist 300 | γH2AX foci levels were 107% higher  in CT patients with ICA |

**References in the Table S1:**

1. Norman A, Adams FH, Riley RF (1978) Cytogenetic effects of contrast media and triiodobenzoic acid derivatives in human lymphocytes. Radiology 129:199-203.

2. Cochran ST, Khodadoust A, Norman A (1980) Cytogenetic effects of contrast material in patients undergoing excretory urography. Radiology 136:43-46

3. Parvez Z, Kormano M, Satokari K, Moncada R, Eklund R (1987) Induction of mitotic micronuclei by X-ray contrast media in human peripheral lymphocytes. Mutat Res 188:233-239

4. Sinues B, Nunez E, Bernal ML, Alcala A, Saenz MA, Conde B (1991) Micronucleus assay in biomonitoring of patients undergoing excretory urography with diatrizoate and ioxaglate. Mutat Res 260:337-342

5. Corde S, Joubert A, Adam JF et al (2004) Synchrotron radiation-based experimental determination of the optimal energy for cell radiotoxicity enhancement following photoelectric effect on stable iodinated compounds. Br J Cancer 91:544-551.

6. Grudzenski S, Kuefner MA, Heckmann MB, Uder M, Lobrich M (2009) Contrast medium-enhanced radiation damage caused by CT examinations. Radiology 253:706-714.

7. Kuefner MA, Grudzenski S, Hamann J et al (2010) Effect of CT scan protocols on x-ray-induced DNA double-strand breaks in blood lymphocytes of patients undergoing coronary CT angiography. Eur Radiol 20:2917-2924.

8. Pathe C, Eble K, Schmitz-Beuting D et al (2011) The presence of iodinated contrast agents amplifies DNA radiation damage in computed tomography. Contrast Media Mol Imaging 6 :507-513.

9. Beels L, Bacher K, Smeets P, Verstraete K, Vral A, Thierens H (2012) Dose-length product of scanners correlates with DNA damage in patients undergoing contrast CT. Eur J Radiol 81:1495-1499.

10. Deinzer CK, Danova D, Kleb B, Klose KJ, Heverhagen JT (2014) Influence of different iodinated contrast media on the induction of DNA double-strand breaks after in vitro X-ray irradiation. Contrast Media Mol Imaging 9:259-267.

11. Piechowiak EI, Peter JF, Kleb B, Klose KJ, Heverhagen JT (2015) Intravenous iodinated contrast agents amplify DNA radiation damage at CT. Radiology 275:692-697.
